# The midwives’ experiences of the use of obstetric triage and obstetric triage tool during labour in Bojanala district

**DOI:** 10.4102/hsag.v27i0.1758

**Published:** 2022-03-31

**Authors:** Kagiso P. Tukisi, Annie Temane, Anna Nolte

**Affiliations:** 1 Department of Nursing Science, Faculty of Health Sciences, University of Johannesburg, Johannesburg, South Africa; 2 Department of Nursing Science, School of Heath Care Sciences, Sefako Makgatho Health Sciences University, Pretoria, South Africa

**Keywords:** initial assessment of labour, experiences, labour, midwife, obstetric triage, obstetric triage tool

## Abstract

**Background:**

Obstetric triage (OBT) is a standardised procedure, which plays a vital role in identifying women with obstetric risks upon admission for labour worldwide. In the last few years, considerable attention has been paid to perinatal problem identification programmes, and it has been determined that the inconsistent use of OBT delays midwives’ responses to both existing and potential clinical problems amongst women in labour. This delay results in negative and serious perinatal outcomes that could have been prevented. This study was conducted to explore and describe midwives’ experiences with OBT in Bojanala district.

**Aim:**

This study aimed to explore and describe midwives’ experiences with OBT in Bojanala district.

**Setting:**

This study was conducted in Bojanala district of the North West Province. Two public healthcare facilities were selected where midwifery care and OBT services are rendered.

**Methods:**

A qualitative, descriptive, explorative research design was followed. Nine purposefully sampled midwives participated in a one-on-one in-depth interview. Data were analysed using Collaizi’s descriptive method based on the themes and categories that emerged.

**Results:**

Three themes emerged. Midwives experienced the OBT tool to be inadequate; and that the low staff number contributes to an imbalance in the midwife–patient ratio. Midwives were also dissatisfied with less support they receive from their management.

**Conclusion:**

The study highlighted midwives’ experiences of the use of OBT, as presented through their lived experiences. The midwives experienced challenges, which hindered them from practicing OBT to the best of their abilities.

**Contribution:**

The study highlighted challenges experienced by midwives regarding OBT, which directly influence the outcomes of pregnancy and labour.

## Introduction

Obstetric triage (OBT) refers to comprehensive assessment using an OBT assessment tool, which includes a series of examinations to evaluate foetal and maternal well-being, assess labour and all other procedures necessary in obstetric management (Ruhl et al. 2020). The Association of Women’s Health, Obstetrics and Neonatal Nurses introduced OBT in 1986 to aid in obstetrical assessment and clinical decision-making (Moudi et al. 2020). Obstetric triage is performed to gather subjective and objective clinical data from a pregnant woman following a nursing process approach to diagnose and manage obstetric conditions upon admission (Veit-Rubin et al. 2017).

Obstetric triage procedures include review of the antenatal history, as well as routine blood investigations for HIV, rapid plasma reagin, Rhesus factor and haemoglobin to exclude anaemia (DOH 2016). A physical examination is performed to assess the woman’s physical, emotional and psychological status related to labour (Mathibe-Neke & Mondell 2017). Vital signs are monitored to exclude pre-eclampsia, gestational diabetes and bleeding because these conditions could be exacerbated during labour, leading to complications such as foetal distress, prolonged labour and postpartum haemorrhage (PPH) (DOH 2016). Abdominal examination is performed to assess the foetus. Uterine contractions are also felt and an initial cardiotocograph tracing needs to be performed to assess foetal well-being (DOH 2016). Vaginal examination is performed to determine the progress of labour (Sellers 2018).

During OBT, clinical judgement is applied as clinical data are collected and meanings are drawn from it in order to make a correct diagnosis. This is usually followed by an appropriate midwifery plan of care that is designed for each individual patient according to urgency and the risk of further complications, which could result in negative perinatal outcomes (Baraki et al. 2017).

Recent evidence from the Saving Babies (2016) report reflected a reduction of 17% in maternal deaths, 48% in non-pregnancy-related deaths, 15% in haemorrhage-related deaths, but 14% increase in pre-eclampsia related deaths. Although there is a marked decline in maternal mortality rates, this analysis has not received general acceptance as conditions, such as HIV, haemorrhage and hypertension, remain significant causes of maternal deaths (accounting for 75% of these deaths), according to the World Health Organization (WHO) (2019) many of these deaths could have been prevented. Perinatal Problems Identification Programme (PPIP) Audits revealed that there is a delay in midwives responding to clinical problems during labour, which results in avoidable negative perinatal outcomes (Rhoda et al. 2018). Furthermore, evidence from the Saving Babies (2016) report revealed that of all perinatal deaths 18% were neonates less than 1000 g, 66.3% were less than 2500 g and in 72% of foetal deaths mothers had no pre-existing medical condition that could have led to intrauterine foetal death. In the light of these findings, intrapartum hypoxia remains a leading cause of neonatal deaths. Furthermore, a PPIP analysis revealed that 53.4% of intrauterine hypoxia-related deaths were medical personnel-related factors (Saving Babies 2016). These deaths were caused by distress in cases where the foetal heart rate was monitored but could not be interpreted, and in some cases, the foetal heart was never monitored; not even during OBT (Saving Babies 2016).

Proper and consistent use of OBT on women admitted for labour results in an improved quality midwifery care – the women in labour are admitted and comprehensively assessed within few minutes of arrival, which limited obstetric complications, thus enhancing women in labour’s safety (McCarthy et al. [in press]; O’Rourke et al. 2018). Previous research failed to address the experiences of midwives with regard to the use of OBT. This study, thus sought to explore and describe midwives’ experiences with OBT in the Bojanala district. In line with the Theory for Health Promotion in Nursing (University of Johannesburg 2017), this study could be a useful aid to midwives to improve their skill and the quality of care provided to women in labour. The findings could also support decision-makers in terms of policymaking regarding OBT and maternity care, which could potentially improve perinatal outcomes.

## Purpose

This study explored and described midwives’ experiences of OBT tool in Bojanala District, North West province.

## Research design

A qualitative, explorative, descriptive and contextual research design was followed to discover the midwives’ lived experiences with OBT in Bojanala district. A researcher asked a central question to all participating midwives and captured their responses as they naturally told their experiences of OBT in Bojanala district. Qualitative research was an ideal method to gain an in-depth knowledge and meaning about midwives experiences.

### Research setting and context

The setting for the study was Rustenburg subdistrict, which is one of five subdistricts in Bojanala district, which comprises 21 clinics, 3 midwife-led obstetric unit (MOU) and one tertiary hospital. One MOU and the hospital within the Bojanala district were used for data collection. The MOU had a population of 11 midwives and there were 23 midwives at the hospital. The MOU and the hospital provide intrapartum care services to the residents in the Rustenburg subdistrict. The hospital under study is the only secondary hospital within the Bojanala district that serves as a referral hospital for maternity cases from the district hospital, MOUs and clinics. The MOU is located in one of the largest townships within the Bojanala district. The hospital accounts for 750 normal vaginal deliveries attended to by 21 midwives, whilst the MOU accounts for 350 deliveries attended to by 11 midwives per month. The above-mentioned factors led to a high influx of patients into the facilities. As a result, the midwives in the hospital and MOU work in resource-constrained environments with a midwife to patient ratio of approximately 1:4.

### Population and sampling

The population of this study comprised all the midwives and advanced midwives employed in two selected facilities providing midwifery healthcare services within the Bojanala district. A purposive sampling technique was used to select midwives and advanced midwives using the following inclusion criteria:

- Employment within the Bojanala district
- Current placement in midwifery unit where OBT is practiced
- A minimum of three years’ midwifery experience
- Current registration with South African Nursing Council (SANC) as a midwife and post-basic qualification in midwifery.

Midwives and advanced midwives who did not meet the given inclusion criteria were excluded from the study, using the following exclusion criteria:

- Non-employment by Bojanala district
- Midwives and advanced midwives registered by SANC not currently in midwifery units
- Less than 3 years of midwifery experience.

### Sampling process

The researcher contacted the participants telephonically using the contact details sent to him by the operational managers. During the telephone call, the researcher inquired from the midwives using the inclusion criteria about their eligibility to participate in the study. In respect of protection of personal information (POPI), the researcher ensured that the provided participants’ contact details were recorded against pseudonyms generated for the study. The list of contacts was kept under lock and key to prevent access to third parties. Twelve midwives who met the inclusion criteria were initially recruited through a research information letter via their operational managers who were identified as gatekeepers for participation in the study. Only nine voluntarily agreed to participate in the study, resulting in a 75% response rate. The researcher contacted the participants telephonically using the contact details sent to him by the operational managers. The researcher used this opportunity to explain the purpose and objectives of the study, asked for participants’ voluntary consent and arranged for the interviews. The participants claimed it would be more convenient for them to be interviewed in their own homes during their day off, as it is difficult to take some time off when they are on duty. The participants thus scheduled appointment dates and times that were convenient for them and the researcher travelled to their respective homes. This choice was acceptable because it allowed the midwives to express themselves freely, away from their place of work.

### Data collection

Data were collected between February and May 2019. The first two participants were considered for a pilot study. The pilot study took place to test if the research question would be understandable to participants and elicit in-depth information about the research topic (Polit & Beck 2017). The analysed data from the two participants confirmed that the midwives were able to express their experiences regarding OBT. Therefore, there was no need to rephrase the research question and these participants were included in the sample of the main study.

One-to-one, in-depth interviews between the researcher and the participants in participants’ homes were conducted. One central, open-ended question was asked from each participant: ‘what is your experience regarding the use of obstetric triage?’ This was followed by probing questions, based on participants’ responses, to ensure in-depth data were obtained. The interviews of participants, which lasted from 30 min to 55 min, were recorded to give the participants ample time to explain their experiences of using OBT. The recordings were transcribed verbatim within 24 h whilst the researcher still had a vivid memory of the interview. The transcribed data were saved in a password-encrypted document and was accessible only to the researcher as a measure to protect participants’ privacy and confidentiality. Data were collected up to a point of data saturation, which was observed after the seventh interview. The data saturation was confirmed by noting repetition of data and non-emergence of new information in the subsequent interviews.

### Data analysis

Collaizi’s seven-step descriptive phenomenological data analysis technique was employed to analyse data (Abalos et al. 2016). This was done with the aim of describing the midwives’ experiences with OBT in Bojanala district by reflecting participants’ words using inductive reasoning (Creswell & Poth 2018). The transcriptions were read intensely to elicit and generate meaning from them. Significant statements were drawn from the interviews to uncover participants’ meanings, which were later clustered into themes for the formulation of exhaustive descriptions of the midwives’ experiences regarding the use of OBT. To ensure bracketing, a researcher admitted that he is an advanced midwife and acknowledged his own assumptions, thoughts and opinions about the topic. The researcher analysed data as expressed by the participants themselves and put aside his own thoughts. The independent coder was employed to confirm the themes and categories. The analysed data were validated by revisiting all participants, who confirmed that the analysed data were descriptive of their lived experiences.

### Trustworthiness

Lincoln and Guba’s framework was employed as a measure to ensure trustworthiness and includes the principles of credibility, transferability, dependability, confirmability and authenticity (1994 cited in Polit & Beck 2017:559). One central open-ended question (followed by probing questions) was posed to the participants who met the inclusion criteria to ensure consistency during all in-depth semi-structured interviews (Polit & Beck 2017:747). The researcher kept an audit trail of each interview by documenting all the information pertaining to the research and initial data analysis (Polit & Beck 2017:560). Furthermore, field notes were written to help formulate clear interpretations of midwives’ statements about their lived experiences regarding the use of OBT. An independent coder was appointed to ensure the validity of the research findings (Polit & Beck 2017:744). The researcher met with the independent coder to discuss both preliminary and final results and a consensus was reached. The research supervisor, who is an expert in qualitative research methodology and analysis, reviewed and accepted the codes. Member checking also took place when all participants were revisited once the data analysis was concluded, and they validated that results were indeed reflective of their own viewpoints and experiences (Polit & Beck 2017:564).

### Ethical considerations

Prior to starting the research process, approval to conduct the study was sought from the University of Johannesburg higher degrees committee, and ethical clearance was obtained from the research ethics committee (reference number: REC- 01-172-2018). Further permission was sought from the North West Department of Health research committee, which approved the research. Permission for access to the Bojanala health facilities was granted by the Bojanala health district research committee and facility managers.

As the research included human subjects as participants, ethical considerations were applied throughout the research process. Therefore, midwives were fairly selected according to pre-determined inclusion criteria to ensure justice, and they were informed of the research purpose and the benefits versus the risks of the study via a detailed research information letter to maintain their autonomy and ensure non-maleficence. Moreover, care was taken to ensure their participation in the study remained confidential, and their identity was protected through the generation of codes specific to the study. All the recordings were stored on a password-protected hard drive and were only accessible to the researcher.

## Results

Data were collected from a sample of seven midwives and two advanced midwives with 5 to 18 years of clinical midwifery experience detailed in Table 1. Three primary themes and related categories were established upon data analysis, reflecting midwives’ experiences of the use of OBT detailed in Table 2.

**TABLE 1:** A description of the participants’ demography.

Code	Age	Gender	Ethnicity	Qualifications	Experience (years)	Facility
P1	27	Female	Black	Bachelor of Nursing and Midwifery	5	MOU
P2	40	Female	Black	Diploma in Nursing and Midwifery	16	Hospital
P3	42	Female	Black	Diploma in Nursing and Midwifery	8	MOU
P4	52	Female	Black	Diploma in Nursing and Midwifery Diploma in Advanced Midwifery	18	Hospital
P5	27	Female	Black	Diploma in Nursing and Midwifery	5	MOU
P6	40	Female	Black	Diploma in Nursing and Midwifery Diploma in Advanced Midwifery	12	Hospital
P7	40	Female	Black	Bachelor of Nursing and Midwifery	16	Hospital
P8	26	Female	Black	Bachelor of Nursing and Midwifery	5	MOU
P9	36	Female	Black	Diploma in Nursing and Midwifery	7	Hospital

MOU, midwife-led obstetric unit.

**TABLE 2:** Summary of the themes and subthemes.

Theme	Subthemes
1. Inadequate OBT tool	Unreliable, not user friendly
2. High patient numbers	Self-referred patients Lack of policies
3. Inadequate management support	Unsupportive management The infrastructure is unsupportive of quality nursing care Shortage of medical equipment and medication Shortage of human resources: midwives Shortage of human resources: doctors

OBT, Obstetric triage.

### Theme 1: Inadequate obstetric triage tool

The midwives experienced the triage tool to be inadequate in alerting them of the extent of the patient’s condition. They expressed that, it is failing mothers and their unborn babies and compromising holistic midwifery care.

#### Category 1: Unreliable, not user friendly obstetric triage tool

The midwives experienced that the current OBT tool’s design made it difficult for them to follow the systematic approach to collect all the relevant clinical data from the women in labour upon admission. As a result, the current OBT tool does not give a clear picture of the patient’s clinical condition:

‘If you look at our document it’s not the one that you can say it’s very good!’. (P4, advanced midwife [AM], at the hospital [H])

‘Initial assessment as it is cannot give you a clear picture of how is the patient’. (P7, midwife [M], H)

Participants verbalised that they found that it lacks some vital aspects in triaging women in labour. This puts the lives of the mothers and their unborn babies at risk, as they are unable to prioritise and promptly respond to patients:

‘This tool is failing our patients actually’. (P4, M, H)

The midwives verbalised that they are limited to comprehensively assess women in labour as the OBT tool does not provide space for all the necessary vital signs to be detailed on admission:

‘I think they should amend the initial assessment of labour especially with the vital data … not all vital data are there like I said, the glucose is not even there you know’. (P5, M, MOU)

The midwives expressed that the other experienced limitation within the current OBT tool is the space for them to record results of rapid investigations they are to perform during OBT such as haemoglobin:

‘There are no spaces to write rapid results such as RH, if you did it, or HB, when you admit the patient you must write the current HB’. (P7, M, H)

The midwives experienced that there is limited space on the OBT tool to allow them to write a comprehensive OBT report, which results in missing patient’s vital data:

‘Right now you will be writing short notes because there is no space’. (P6, AM, H)

The midwives experienced that they were limited in writing a comprehensive report of their OBT clinical findings, diagnosis and care plan, which are the pillars of OBT:

‘I would like them to give us more space to write how the patient was on admission … and the management as it is important’. (P6, AM, H)

‘[*T*]here is no space for it even when you go to management, there is a lot to write about, we must plan and manage so I don’t know how we can manage the patient without planning for the patient’. (P7, M, H)

Midwives reiterated that the lack of space interferes with the interprofessional communication and continuity of care to be received by the woman in labour when transferred from one unit to another or from one facility to another:

‘The space is so limited’. (P3, M, MOU)

‘When the patient is transferred to another level they don’t have full information’. (P6, A M, H)

‘[*Y*]ou need to elaborate on what you have done and how you have done it so that you are able to explain to another person who will be holding the record without you being there and saying it verbally to their face’. (P5, M, MOU)

Midwives insisted that they are the users of the OBT tool, and they should be the ones responsible for its revision for it to be holistic and reliable because as it is not user-friendly:

‘This tool needs to be revised and … my suggestion is that they should call midwives’. (P4, AM, H)

‘Our current tool is not user friendly at all’. (P1, M, MOU)

### Theme 2: High patient numbers

The midwives experienced that the high influx of patients contributes to medical and obstetric-related complications. The midwives associated the high influx of patients with the uncontrolled entry of self-referred patients because there are no admission policies in place.

#### Category 1: Self-referred patients

The midwives seemed to be aware that their experienced high influx of patients in their midwifery units is related to a rapid increase in population in Bojanala district:

‘The Bojanala district, there is a very large population that are coming to work in the mines; there are a lot of pregnant ladies’. (P9, M, H)

The midwives experienced that pregnant women in labour arrive at the hospital without being referred, which causes a high influx of patients because the midwives have no control over their movements. They cannot deny the women services or redirect them to relevant levels of care because of the laws of the country pertaining to health:

‘Self-referrals with no problems they just pop in’. (P2, M, H)

‘You are having low-risk patients but you cannot take them back, so there is no down-referral policy’. (P5, M, MOU)

The midwives shared that this caused an interruption in their work because they were obliged to perform OBT and admit such low-risk patients. This caused an overflow of patients taking up space meant for high-risk patients:

‘Patients who should have delivered at the clinic end up coming to the hospital and patients who need hospital care end up sleeping on the floor’. (P4, A M, H)

#### Category 2: Lack of policies

Clinical findings from OBT aid in women in labour’s classification as either low or high risk. Midwives are thus required to devise an appropriate plan of care based on these findings. They also determine where such care will be rendered and refer the women to appropriate levels of care (DOH 2016). Participants were aware of this expectation:

‘Patients should be referred from the clinic because they need specialist care’. (P8, M, MOU)

‘They say our hospital is a tertiary institution and we are only supposed to take the patient from hospitals but we are taking … self-referrals that are unbooked’. (P7, M, H)

Participants shared that they have no control over women being admitted in the OBT units because of a lack of well-defined admission and down-referral policies; this forces them to function with limited protection. A high influx of self-referred women ensues, whom they are supposed to admit despite being low risk and eligible for referral to the lower levels of care, as per the OBT findings.

As a result, they end up with more patients than they can handle, placing them and their patients at risk:

‘In our institution we don’t have any policies … there are no written policies’. (P8, M, MOU)

‘They are saying we must down refer the patients but it’s very difficult when you don’t have the policy that is covering you!’. (P6, A M, H)

Midwives associated their lack of control over women’s movement in and out of their facilities as the source of overcrowding and imbalance in midwife to patient ratios. They explained that low-risk patients end up taking space meant for high-risk women:

‘Patients who should have delivered at the clinic end up coming to the hospital and patient who needs the hospital care ends up sleeping on the floor’. (P8, M, MOU)

Participants mentioned that the doctors with whom they are working in OBT rooms also find it difficult to manage the admission of women in labour without proper policies. Accordingly, even low-risk women become part of those managed in the high-risk labour ward:

‘Even in the admissions the doctors who are working don’t know the policies. As long as they say the patient is pregnant they will admit’. (P7, M, H)

Moreover, without well-defined policies, midwives experienced that care plans at the end of OBT are non-standardised. Thus, specific care depends on the prescribing doctors’ preference for each case:

‘There is no policy, when the previous caesaereans come in labour it depends on the doctor’. (P4, AM, H)

### Theme 3: Inadequate management support

The midwives were dissatisfied with management, who did not provide sufficient human and material resources. Midwives were frustrated with the management team, which seemed to overlook the serious shortage of staff, equipment, drugs and proper infrastructure. They related these shortcomings as hindering appropriate OBT of women in labour, which result in negative perinatal outcomes.

#### Category 1: Unsupportive management

Midwives expressed that the unfavourable conditions in which they are working left them overburdened and burnt out, slowing their productivity in the OBT units. Despite management’s awareness of the midwives’ situation, they appeared unsupportive and uncaring; this left the midwives feeling dissatisfied and angry:

‘People are burnt out because there is nobody who cares about the carer these days’. (P4, A M, H)

‘Even the management – they don’t support us because even if they see you are there on duty, they don’t see it as long as you push the work they are fine’. (P7, M, H)

Participants explained that despite their attempts to render midwifery and the related OBT care, under what they described as ‘unfavourable circumstances’, they received no appreciation from their superiors, which left them angry and discouraged:

‘If you are not appreciated by your supervisors it makes it even more difficult for you to love what you do … you are always scared that should something happen here I am going to be crucified instead of being supported’. (P8, M, MOU)

#### Category 2: The infrastructure is unsupportive of quality nursing care

Midwives experienced that their health facilities are not sufficient for the communities they serve. They expressed the need for additional facilities to ease their burden:

‘We really need specific hospital for maternity’. (P4, AM, H)

Participants shared that with the high influx of patients and lack of admission policies to control inpatient movements, there is not enough physical space in the obstetric units for admissions and inpatients. This is detrimental to the quality of midwifery and related OBT care:

‘There is no space; we will be having only two beds for admission, so whilst you are admitting this one … they are on the bench waiting on the queue, waiting for you to admit them’. (P3, M, MOU)

Midwives experienced that the lack of space in their facilities compromised women’s right to privacy and confidentiality, as women have to share delivery rooms divided only by a curtain. This set-up allows one woman to see the other woman and overhear conversations:

‘There is no privacy in our labour rooms, because you will find that there are two women in one labour room, and then when you are delivering this one and you close the curtain!’. (P6, AM, H)

#### Category 3: Shortage of medical equipment and medication

Obstetric triage requires the use of specific equipment, such as haemoglobin monitors. Midwives experienced that management seem to overlook the serious shortage of working and available equipment. They stated that this often leads to a negative outcome for mothers and babies, as they are unable to get accurate clinical findings during OBT-specific procedures:

‘We also have a problem with equipment’. (P3, M, MOU)

‘The HB meters, how can you check an HB when you don’t have an HB meter? We cannot test glucose, no urine sticks. So how are we managing patients?’. (P4, AM, H)

Participants were thus concerned about the validity and accuracy of their OBT findings, as the equipment in use is unreliable, and this could lead to misdiagnoses. They expressed that there is also no service plan in place for the limited equipment available to them to ensure they continue to function optimally:

‘Our machines are working non-stop … the machine take time to be serviced at times you are not so sure if the readings that they are giving are what is happening with the patient’. (P3, M, MOU)

According to the participants, they were also experiencing severe shortages of life-saving equipment (such as oxygen points) that is essential for interventions in obstetric emergencies:

‘And also we have got a problem with oxygen points, we only work with one. Sometimes its three [*women with*] foetal distresses, you don’t know where to put the patient on oxygen because there is only one point’. (P7, M, H)

#### Category 4: Shortage of human resources: midwives

Midwives shared that the high influx of patients was more than the staff could manage. As a result, they often found themselves being alone in admissions, with the responsibility of too many patients. The midwives expressed that this is a source of negative perinatal outcomes.

‘My biggest challenge is shortage of staff’. (P3, M, MOU)

‘You find that one midwife is working in admission and also fetching caesarean sections’. (P4, AM, H)

Midwives experienced that an imbalance in the midwife to patient ratio, where one midwife is responsible for a high number of patients, resulted in a serious decline in the quality of midwifery care being rendered during OBT:

‘So due to this influx, there is no proper assessment’. (P4, AM, H)

‘You will not be able to… prioritise your patients accordingly … So this is also causing the…delay in taking action on the complicated patients’. (P6, AM, H)

It was also reported that it is impossible for midwives to speedily attend to women in labour upon admission. This delay resulted in an increase in women’s waiting time for OBT:

‘One assessment per patient it takes thirty [*minutes*] … Meaning that the time for me to see the patient is going to be delayed by thirty minutes, sometimes it takes more’. (P9, M, H)

With the delay in care due to an increase in waiting time and workload, the midwives experienced that the women’s conditions became worse and some even delivered unattended whilst waiting for triage:

‘As the workload increases the time is delayed as to when I see patients that really need that care’. (P9, M, H)

‘And some will deliver on those chairs’. (P2, M, H)

Participants shared their value for teamwork and its importance in the OBT areas but experienced that teamwork is difficult to accomplish with low staff numbers:

‘The sister working in the labour room is alone … and the other one working in admission is alone’. (P2, M, H)

‘Without teamwork patients are going to suffer’. (P3, M, MOU)

#### Category 5: Shortage of human resources: doctors

Participants reported that the shortage of staff is not only affecting the midwifery teams but also the doctors who form part of their multidisciplinary teams in the OBT setting:

‘I think we have shortage of doctors … We are hammering about that in triage’. (P3, M, MOU)

With the shortage of doctors, midwives experienced that they often find themselves teamed up with junior doctors who are supposed to design advanced obstetric plans of care for high-risk patients following triage. However, because of the complexity of some of the obstetric conditions, it is often difficult for junior doctors to accurately diagnose and manage such patients:

In triage you will find that we are allocated with an intern or community service doctor [*sic*]. (P4, AM, H)

‘Sometimes they don’t know what to do because they don’t have experience’. (P7, M, H)

The midwives advocate for patients as far as obstetric plans of care are concerned within the multidisciplinary team. They therefore experienced that teaming up with junior doctors under these circumstances is a challenge and compromises the multidisciplinary team relationships, as they are sometimes obliged to assist the doctors in deciding on plans of care, leaving the doctors feeling undermined:

‘Sometimes the doctors feel that they can’t be reprimanded or they can’t be told what to do by the midwives’. (P8, M, MOU)

‘So now your triage is useless because their diagnosis comes first’. (P4, AM, H)

The shortage of staff also compromised inter and multidisciplinary teamwork, leaving midwives to be responsible for an even larger number of patients in their care. This led to feelings of burnout and a lack of care:

‘We are so overworked’. (P3, M, MOU)

‘People are burnout because there is nobody who cares about the carers these days’. (P8, M, MOU)

## Discussion

The study explored and described midwives’ experiences with OBT in Bojanala district. The findings that emerged from the data were remarkable as midwives seemed aware of the importance of OBT and its usefulness in ensuring the safety of mothers and their unborn babies by identifying and anticipating problems and planning care for them. They demonstrated a willingness to render this service but were troubled about the challenges surrounding the OBT, which led to inconsistencies in its use.

The findings of this study revealed that the OBT tool in use is inadequate for detailed clinical assessment and proper classification of women in labour upon admission. These results substantiate the findings from a study on PPIP by Allanson, Muller and Pattinson (2015:3), which recorded 150 stillbirths and 271 early neonatal deaths, of which a majority could have been avoided. The most conspicuous finding was that the impending danger and associated risks could not be predicted in any of these recorded deaths. In an analysis by Hasegawa et al. (2019) of maternal deaths because of obstetric haemorrhage, a significant decline in maternal deaths was experienced where the incidence of a haemorrhage was anticipated upon admission following a thorough OBT of the woman in labour and appropriate plans of care.

Participants shared their dissatisfaction with the current OBT tool, which prevented them from getting a full clinical picture of a woman upon admission and during handing over for continuity of care. This is encouraging as it shows that the midwives value clinical record-keeping and regard it as an important aspect of their midwifery practice. Literature regarding documentation in nursing care support this finding and suggest that notes should be sequential, detailed and narrative of the patient’s condition and the plan of care being implemented. This is essential to prevent poor communication amongst healthcare professionals (Selvi 2017).

The findings revealed that the national legal and ethical framework pertaining to health and national guidelines on maternity care are contradictory. Midwives seemed aware of the legal and ethical frameworks governing their practice and reiterated that every patient has a right to access healthcare and basic antenatal care mandates that the healthcare facility should be within a 5-km radius for pregnant women to access in case of emergencies to improve positive birth experiences. However, the maternal healthcare system in South Africa is dependent on the referral system (WHO 2016) from lower levels of care to the higher levels of care (DOH 2016). Midwives expressed that they found this contradiction of laws to be limiting their control over patients’ movement according to the maternity care guidelines. Furthermore, this concurs with more recent evidence from a study in Australia, which determined that midwives find the referral system to be beneficial in ensuring safe births. Women with low risk are seen by the midwives, and care plans for those with potential or existing risks such as pre-eclampsia are discussed with doctors for more advanced interventions and referrals (Styles, Kearney & George 2020). This highlights a need for the DOH to ensure consistency in information reaching the public and in the prescribed guidelines for midwifery units.

The correlation between midwives’ inability to control women’s movement in the admission ward following triage, and the imbalance in the midwife to patient ratio is a finding worth noting. Midwives revealed that they often find themselves dealing with more women in labour than they can handle. This leaves the midwives with a major responsibility of caring for these women out of fear of redirecting them to specific healthcare facilities suitable for their level of care and contravening their rights to access healthcare. This imbalance in the midwife to patient ratio makes midwifery and OBT impossible to accomplish, which bears serious consequences. Overcrowding and low staff numbers can result in women’s waiting times in the OBT areas being between 15 min and 100 min, with a maximum of up to one full day (Floyd et al. 2018). In support, Marobwa et al. (2019) reported that women with high-risk conditions such as pre-eclampsia often complicate in instances where they need to queue for assistance, thus causing negative perinatal outcomes. On a positive note, the midwives understand the detrimental effects of increased waiting times to triage, and this could be used as a point of departure in reducing waiting time in the OBT.

Midwives expressed that they are short-staffed and often left to manage emergencies such as eclampsia and cord prolapse that require concerted team efforts in fewer numbers. This finding was validated by the recent analysis of midwives’ experiences that staff shortage makes them feel overwhelmed with emergencies (Matlala & Lumadi 2019). Moreover, although the shortage of doctors is a known scarcity in South Africa – as per the recent report by Ntsaluba (2019) – the country has been unable to employ many newly qualified doctors. Midwives therefore revealed that they often team up with inexperienced doctors who are unable to manage most conditions based on OBT findings, causing further delays. Barnawi et al. (2017), who claimed that junior doctors are able to follow the prescribed procedures on history taking and physical examination, reported a similar phenomenon yet their records of these activities are often incomplete, which leads to inaccurate diagnoses.

Regrettably, this study found that the multidisciplinary team member relationships between midwives and doctors were strained during OBT. Midwives expressed that they find it difficult to reason with junior doctors who sometimes do not value their opinion as far as plans of care are concerned, causing miscommunication and delays in life-saving interventions. Doctors and midwives are supposed to team up and be at the forefront in OBT settings to immediately diagnose, plan and implement necessary interventions. Unfortunately, this is not the case, leaving midwives feeling demoralised and devalued by the doctors (Matlala & Lumadi 2019).

It was overwhelming to find out that despite management’s awareness of the midwives’ long-standing challenges in the OBT; they had failed to provide material resources and drugs necessary for OBT procedures. Midwives expressed that they sometimes do not have basic equipment such as cardiotocograph machines to monitor foetal heart rates on admission. This lends support to McMicking, Vieira and Pasupathy (2020) argument that a majority of Intrapartum deaths could be avoided if women receive better care. In addition, midwives mentioned that they often lack important medications, which are to be administered to manage emergencies during OBT. This finding is in line with those of Morton et al. (2019), who claimed that in order to prevent maternal death from eclampsia and PPH, the diagnosis of such conditions should immediately be followed by the administration of necessary life-saving antihypertensive medications, oxytocics, as well as emergency transfusions. Despite management’s awareness of these existing challenges, they have failed to address them, leaving the midwives feeling unsupported, discouraged, with no sense of control over their experiences. This reduced their productivity whilst providing comprehensive OBT services, substantiating Filby, McConville and Portella’s (2016) claim that a lack of management supports is demotivating to midwifery teams.

## Conclusion

This study explored and described midwives’ experiences with OBT in Bojanala district. It was determined that in Bojanala, this critical, life-saving practice was met with several challenges, potentially increasing negative perinatal outcomes. The findings of this study were explored and described using a qualitative and descriptive research design.

The findings of this study indicated that participants generally experienced the use of OBT to be challenging. The results substantiate and endorse findings from existing literature on avoidable factors contributing to negative perinatal outcomes. The considerable insights gained on the use of OBT led the researcher to recommend that challenges be addressed in order to enhance consistency in the use of OBT to ensure positive outcomes of pregnancies.

One of the strengths of the study was the identified gaps with the OBT tool from the midwives, which are in line with the existing literature. This has shown that midwives are able to integrate theory and clinical midwifery practice. The limitations of this are as follows: the study was limited to public health facilities in Bojanala district and was not extended to private sector. Midwives were the only participants; doctors are also OBT practitioners and could have shared valuable information.
